# What visual illusions teach us about schizophrenia

**DOI:** 10.3389/fnint.2014.00063

**Published:** 2014-08-12

**Authors:** Charles-Edouard Notredame, Delphine Pins, Sophie Deneve, Renaud Jardri

**Affiliations:** ^1^Pediatric Psychiatry Department, University Medical Centre of LilleLille, France; ^2^SCA-Lab, PSYCHIC Team, Université Lille Nord de FranceLille, France; ^3^Group for Neural Theory, INSERM U960, Institute of Cognitive Studies, École Normale SupérieureParis, France

**Keywords:** illusions, schizophrenia, psychosis, visual perception, hallucinations, delusions, Bayesian inference, predictive coding

## Abstract

Illusion, namely a mismatch between the objective and perceived properties of an object present in the environment, is a common feature of visual perception, both in normal and pathological conditions. This makes illusion a valuable tool with which to explore normal perception and its impairments. Although still debated, the hypothesis of a modified, and typically diminished, susceptibility to illusions in schizophrenia patients is supported by a growing number of studies. The current paper aimed to review how illusions have been used to explore and reveal the core features of visual perception in schizophrenia from a psychophysical, neurophysiological and functional point of view. We propose an integration of these findings into a common hierarchical Bayesian inference framework. The Bayesian formalism considers perception as the optimal combination between sensory evidence and prior knowledge, thereby highlighting the interweaving of perceptions and beliefs. Notably, it offers a holistic and convincing explanation for the perceptual changes observed in schizophrenia that might be ideally tested using illusory paradigms, as well as potential paths to explore neural mechanisms. Implications for psychopathology (in terms of positive symptoms, subjective experience or behavior disruptions) are critically discussed.

## Introduction

Vision has long been considered one of the main routes humans use to understand the world (Glezer, 1995). However, recent findings encourage moving beyond this presupposed primacy of vision over other senses, notably, when looking at the details of visual misperceptions. Visual arts provide eloquent illustrations of common mistakes made in vision. A first example is when artists use artifices to mislead perception and induce, for example, a paradoxical “realistic” feeling of depth or movement. Illusionists also frequently resort to human perceptual properties in their magic tricks (Martinez-Conde and Macknik, 2007) to the extent that the study of these tricks has become the object of an original sub-field in cognitive sciences called “neuro-magic.”

**Figure 1:**
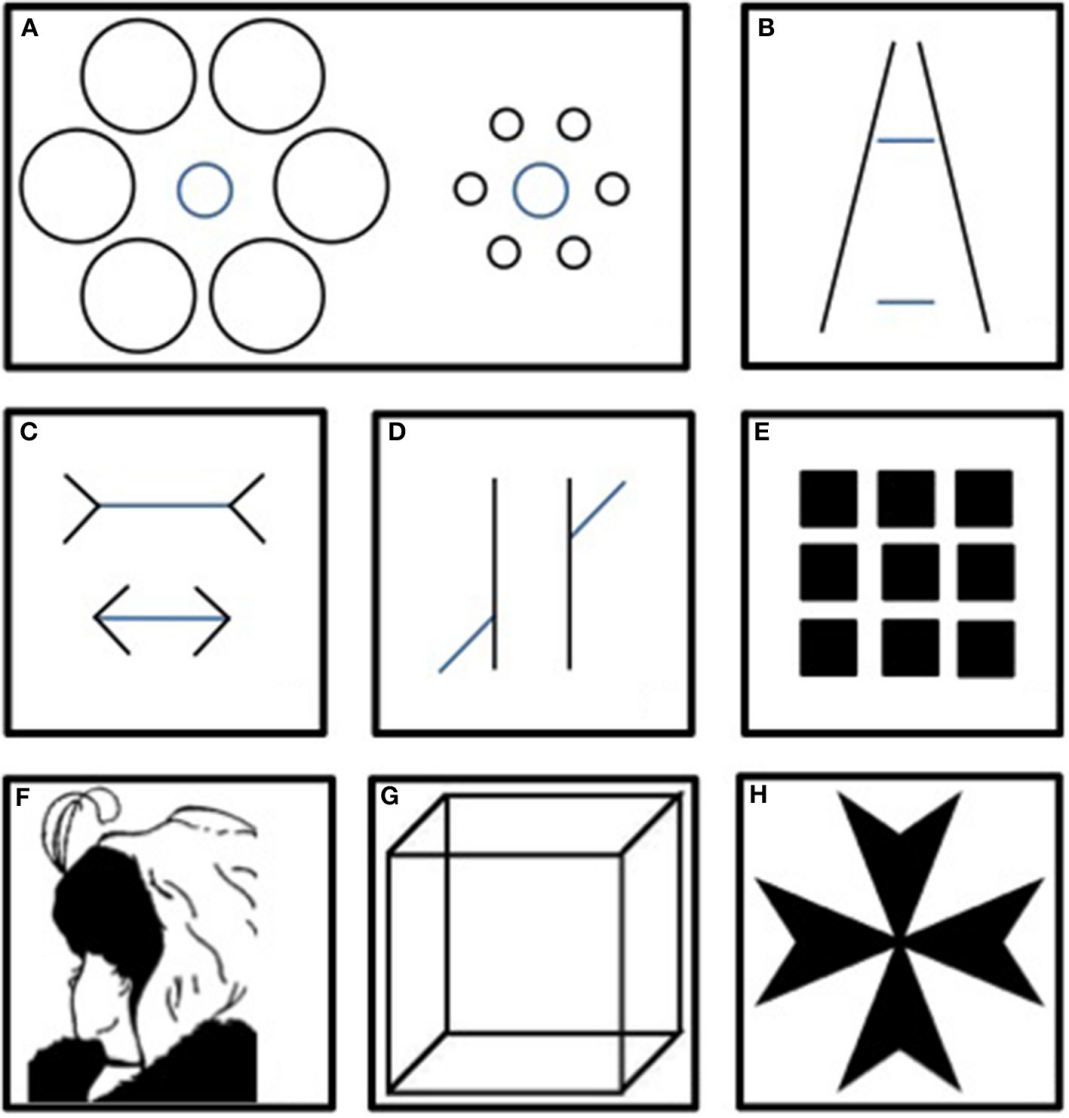
**Main classical illusions**. In the Ebbinghaus **(A)**, Ponzo **(B)** and Müller-Lyer **(C)** illusions, same-sized patterns are misevaluated because of the context. In the Poggendorff illusion **(D)**, the context disrupts the impression of continuity. Herman's grid **(E)** generates illusory gray points at each intersection of the white lines. The Boring wife/mother-in-law **(F)**, the Necker Cube **(G)** and Rubin's Maltese Cross **(H)** are ambiguous figures that result in different interpretations.

Historically, the first scientific descriptions of misleading visual effects date back to the late 19th to early 20th century. Physiologists, such as Poggendorff, Herman, Müller-Lyer, Ponzo and Ebbinghaus, noticed that our appreciation of contrast, size or continuity can be distorted by contextual information (Zölner, 1860; Hermann, 1870; Müller-Lyer, 1889; Ponzo, 1910). Similarly, Necker and, later, Boring and Rubin described ambiguous figures that could lead to different, mutually exclusive interpretations (Necker, 1832; Boring, 1930; Rubin, 1958) (See Figure 1). These visual effects were named visual illusions (VIs) or optical illusions. Progressively, VIs assumed a place of increasing importance in the literature as a fertile, practical and valid way to explore the underlying mechanisms of perception in normal or pathological conditions.

There is a strong tradition of considering as illusory every image that misleads perception into instability, insolubility, distortion or fiction (for an example of synthetic classification, please refer to Table 1). As a consequence, an overview of the literature reveals that the stimuli traditionally considered as illusory vary greatly in terms of (a) complexity [from the simple *Three flash illusion* (Bowen, 1989) to the more ecological *Hollow-mask illusion* (Gregory, 1973)]; (b) the perceptual mechanisms or physiological pathways involved (e.g., contrast vs. motion illusions); (c) the level of integration required (e.g., contrast detection vs. bistable perception induced by an ambiguous figure); and (d) the type of subjective impression that they may induce (e.g., apparition of fictive gray points in the *Hermann's grid* vs. distorted size perception in the *Ebbinghaus illusion*). Such diversity is actually consistent with the important difficulty to consensually define illusions. On a purely theoretical level, authors such as Gregory (1997a) and Eagleman (2001) noted the pitfall of simply considering illusions as a gap with reality, a definition that might correspond to the whole process of perception. In contrast to this extensive point of view, an excessively restrictive definition would risk characterizing sub-categories of illusions rather than the general perceptual phenomenon. This would consequently impede any theorization. As a starting point for this review, we opted for the classical compromise of considering illusions as a systematic mismatch between the basic response of the sensory organs to a stimulus (related to its physical properties), and the percept this object gives rise to. Nevertheless, this working hypothesis urges a theoretical clarification, which the Bayesian framework will help us to address in Section Visual Illusions and the Bayesian Theory.

**Table 1:** **Gregory's classification**.

**Classes**	**Example**	**Description**
Ambiguity	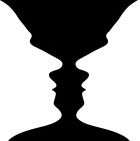	Information is insufficient to result in a single interpretation.
		**Rubin's figure** can be perceived either as a vase (black) or as two face-to-face characters (white).
Distortion	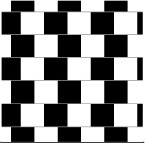	The visual context induces a distortion in size, contrast, motion or disposition appreciation.
		In the **Café Wall illusion**, the lines, although parallel, appear to be convergent or divergent.
Paradox	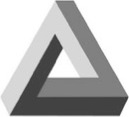	The figure appears to be an impossible object when viewed from a critical position.
		The **Penrose triangle** introduces a “mise en abyme,” which makes the figure implausible.
Fiction	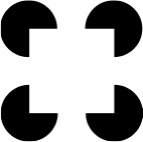	The observer perceives visual elements absent in the figure because of the context.
		The **Kanisza square**'s contour is reconstructed by the perceptual system.

*The classification of visual illusions (VIs) highlights the difficult problem of criteria choice. In one of his categorizations, Gregory empirically chose to cluster VIs on the basis of an analogy between their appearance and the main language errors (e.g., ambiguity, distortion, paradox and fiction) from which he assumed they were derived (Gregory, 1997b)*.

In this perspective, illusions, hallucinations and hallucinosis are experiences that share the property of being inconsistent with the actuality of the sensory environment. As such, they all belong to the category of false percepts. Nevertheless, the present review is based upon crucial points of the definition in order to distinguish illusions from other misperceptions. VIs originate from an object already present in the environment and occur frequently in “normal” visual processing, whether naturally induced or intentionally provoked. The vulnerability to VIs does not have any pathological significance *per se*, but rather, it can be considered a common phenomenon. In contrast, hallucinations and hallucinosis consist of perceptions without any sensory substratum. Hallucinations can occur in a wide range of conditions, from non-clinical groups to full-blown psychosis. The pathological nature of these experiences basically depends on the strength of the associated beliefs and the extent to which they give rise to delusional interpretations. This point of view posits the existence of a continuum from total confidence, where the hallucinatory percept is completely integrated in the subject's life, to total distrust, where the false percept is felt as odd and interpreted as abnormal (this later end of the continuum characterizes hallucinosis).

These particular precisions are important to understand how the abnormal patterns of illusory perception potentially identified in patients who suffer from schizophrenia differ from, but can be linked to, the other false percepts (e.g., hallucinations). Schizophrenia is a severe and disabling disorder that affects approximately 1% of the general population (McGrath et al., 2008). Although no clear pathophysiological mechanism has emerged to explain the disease, complex impairments in integrative functions are thought to result from a large-scale dysconnectivity syndrome (Frith and Done, 1988; Friston, 1998, 2005a; Burns et al., 2003; Liang et al., 2006; Stephan et al., 2009; Schmitt et al., 2011; Amad et al., 2014). This disturbance would result in aberrant concept formation, delusions, and hallucinations.

In a recent paper, Silverstein and Kean remind us that visual sciences have been invested since the 1950's to improve insight into the brain functioning of schizophrenia patients (Silverstein and Keane, 2011). In line with the assumption that perception is closely interwoven with other high-level functions (such as belief genesis or reasoning; Fletcher and Frith, 2008), several authors have defended the idea that visual perception plays an important role in the psychopathology of schizophrenia (Butler and Javitt, 2005), and constitutes a unique way to explore the underlying mechanisms of reality construction (Silverstein and Keane, 2011). VIs were an early component of visual research in schizophrenia (see first referenced studies: Letourneau and Lavoie, 1973; Letourneau, 1974). More recently, these procedures have gained a renewed interest, with Dakin et al. (2005) as the first authors to postulate a *decreased* susceptibility to illusion in this disorder. This hypothesis relies on a simple paradigm: the participants had to compare the contrast of two patches successively presented. The first patch was isolated or surrounded by a high-contrast annulus, and the second patch was an isolated reference patch. By manipulating the reference-contrast, the authors noticed that the patients who suffered from schizophrenia were significantly less biased by the surroundings and were thus more accurate in contrast discrimination compared with the healthy and psychiatric controls. The capacity of schizophrenia patients to outperform normal subjects in tasks that involved VIs paved the way for a new approach to explore perceptual processing in psychosis. Indeed, it allows controlling for one of the main confounding factors in this population, i.e., decreased performances due to a global cognitive deficit.

In this paper, we review recent VI experiments conducted in schizophrenia patients, with the aim of explaining how apparently composite findings may offer a holistic comprehension of visual perception in schizophrenia. Furthermore, we critically discuss how probabilistic theories of perception (e.g., Bayesian) are of interest to understand the singular pattern of illusion sensitivity in schizophrenia. We also demonstrate how the computational hypotheses of psychosis benefit from VIs to provide insights in the genesis of the positive symptoms and in other psychopathological properties in this disorder.

## Visual illusions: a way to probe the integrity of visual processing in schizophrenia?

### Discussing the limits of the structural approach

The physiological existence of VIs suggests that deceiving perception paradoxically requires the integrity of the different subcomponents of the visual system. At first sight, the corollary assumption appears to make sense: one could consider resistance to VIs as the expression an identifiable, specific and common disruption that affects the structures visual processing. However, trying to synthesize the studies that adopted this structural perspective led to two main limitations.

First, the structural approach conceptualizes the impairments of visual processing as potentially resulting from dysfunctions of the brain structures computing low-level sensory information. If this hypothesis is correct, this would result in poorer experimental performances of the patients compared with controls. To date, the findings based on VI paradigms in schizophrenia do not allow clear-cut conclusions. On the one hand, many authors, following Dakin, reported that patients who suffer from schizophrenia outperform controls in VI experiments. Dakin's findings were notably replicated using the same type of context-based illusion paradigms (Uhlhaas et al., 2004; Tadin et al., 2006; Chen et al., 2008), but were also extended to other VI categories, such as the Binocular Depth Inversion (Koethe et al., 2009), illusory motion and motion-induced illusions (Tschacher et al., 2006; Crawford et al., 2010). On the other hand, some studies evidenced unchanged, augmented or contrasted patterns of sensitivity to VIs in schizophrenia. A summary of the main empirical findings regarding VIs and schizophrenia is provided Table 2. Importantly, most of the negative or nuanced results referred to surround-suppression illusions. This paradigm appears exposed to specific methodological issues and covariates that may be imputed in the results (see Section A Window Into the Functional Dimension of Visual Perception). Even if these data should still be interpreted with caution, the hypothesis of a diminished sensitivity to VIs in schizophrenia appears to be a most robust finding. From a methodological point of view, increased performance in patients cannot be attributed to general task difficulty, a lack of attention or motivation or to a disease-related global deficit. Rather than the classical cognitive deficit-model proposed in schizophrenia, these findings suggest a perceptual process different in nature compared to the one observed in the general population.

**Table 2:** **Schizophrenia, pro-psychotic states and VIs: main empirical findings**.

**Study (year)**	**Condition (sample size)**	**Paradigm**	**Significant findings**
**STUDIES THAT IDENTIFIED A DECREASED SENSITIVITY TO VIS IN SCHIZOPHRENIA OR PRO-PSYCHOTIC CONDITIONS**			
Barch et al., 2012	SCZ-T (132)	Surround suppression (contrast discrimination)	The importance of the surround effect in patients was reduced compared with CTL. *The difference did not survive the integration of the attentional variables into the analysis*
Bressan and Kramer, 2013	Non-clinical population (123)	Surround suppression (size discrimination)	*The size illusion magnitude decreased with cognitive-perceptual schizotypal traits and magical ideations. This reduction was entirely mediated by judgment time*
Crawford et al., 2010	SCZ-T (21)	Apparent motion	The estimated total strength of the illusion was less important in patients compared with controls
Dakin et al., 2005	SCZ-T (15)	Surround suppression (contrast discrimination)	The contrast-contrast effect was diminished compared with both the healthy and psychiatric control groups
	Psychiatric controls (20)		
Emrich et al., 1997	Volunteers exposed to Δ9-THC (7), SCZ (12)	Binocular Depth Inversion Illusion	Patients were more resistant to the illusion compared with CTL. For intoxicated volunteers, the strength of the illusion was negatively correlated with the plasma levels of Δ9-THC
Keane et al., 2013	SCZ and SCZ-T (30)	Binocular Depth Inversion Illusion	Patients were less vulnerable to the illusion compared with CTL. *This resistance was positively correlated with the need for a structured treatment and with positive symptoms*
Koethe et al., 2006	Volunteers exposed to Δ9-THC (16), FEP (16), IPS (16)	Binocular Depth Inversion Illusion	Vulnerability to the illusion was more important in CTL compared with the other groups. *The Δ9-THC-induced psychological manifestations most resembled those of IPS, with thought disorders being the most prominent symptom*
Koethe et al., 2009	SCZ (75), SCZ-T (75), IPS (22), MDD (35), BD (22), Alzheimer's (6)	Binocular Depth Inversion Illusion	SCZ, SCZ-T and IPS tended to be less prone to the illusion compared with CTL. The difference between the other groups and CTL did not reach significance. There was no difference between the clinical groups. *No relevant correlation between the illusory effect and psychopathology was found in any group*
Leweke et al., 1999	Volunteers exposed to Δ9-THC (17)	Binocular Depth Inversion Illusion	BDII was strongly reduced after Δ9-THC administration
Must et al., 2004	SCZ (20)	Surround suppression (Facilitation effect of collinear flankers)	Collinear flankers had a smaller facilitation effect on contrast detection in SCZ compared with CTL. *No correlation was found with clinical symptoms*
Robol et al., 2013	SCZ and SCZ-T (18)	Contour detection + Surround suppression	The contour detection was poorer and less susceptible to the influence of the surround effect in patients compared with CTL. Patients were also less affected by the influence of distractors in discriminating the orientation of contour elements. *In some conditions*, *flankers had less influence on patients who presented with more negative symptoms*
Sanders et al., 2013	SCZ and SCZ-T (34)	Apparent motion	Susceptibility to the illusion was significantly weakened for patients. *The strength of the illusory movement was inversely correlated with delusional belief conviction scores*
Schallmo et al., 2013	SCZ (28), First-degree relatives of SCZ patients (15)	Contour detection + Surround suppression	Contour detection was impaired in patients. Context caused less of a performance decrement in patients compared with CTL or relatives
Schmeider et al., 1996	SCZ (13), patients with alcohol withdrawal (10), Sleep-deprived volunteers (10)	Binocular Depth Inversion Illusion	SCZ and sleep-deprived volunteers were significantly less vulnerable to the illusion compared with CTL
Schneider et al., 1996	Patients with alcohol withdrawal (10),	Binocular Depth Inversion Illusion	Patients were highly more resistant to the illusion compared with controls
Schneider et al., 2002	SCZ-T (10), MDD (10)	Binocular Depth Inversion Illusion	The SCZ group was significantly less vulnerable to the illusion compared with both CTL and MDD during the first week of admission. Before the patients' discharge, the difference was not significant. A trend to resist the illusion was found in the MDD group but did not reach significance. *The strength of the illusion correlated with the Brief Psychiatric Rating Scale scores*
Semple et al., 2003	Chronic cannabis users (10)	Binocular Depth Inversion Illusion	Cannabis users were less prone to the illusion compared with CTL, irrespective of the time since the last dose (which suggests the effects of chronic use). *No correlation was found between resistance to the illusion and psychoticism*
Silverstein et al., 2013	FEP (16), SCZ-T (21)	Surround suppression (size)	At hospital admission, the SCZ group was less biased by the context compared with the FEP and CTL groups. At hospital discharge, vulnerability to the illusion was comparable for the three groups. *Positive, depression and excitement symptoms were positively correlated with context sensitivity in the SCZ group*
Sternemann et al., 1997	Sleep-deprived volunteers (10)	Binocular Depth Inversion Illusion	The strength of the illusion was negatively affected by sleep deprivation
Tadin et al., 2006	SCZ-T (16)	Surround suppression (motion discrimination)	Center-surround interactions were weaker in SCZ-T compared with CTL. This led to greater performance in motion discrimination of large high-contrasted stimuli. *This weakening was positively correlated with negative symptoms*
Tschacher et al., 2006	SCZ-T (34)	Motion-induced blindness	The scores designed to reflect the strength of the illusion were higher in CTL compared with SCZ-T. *The scores were positively correlated with positive and excitement symptoms but negatively correlated with depression factors*
Uhlhaas et al., 2004	Schizotypy (32)	Surround suppression (size) + contour detection	No impairment in visual context processing was found to be related to schizotypy overall. A subset of thought-disordered schizotypal participants demonstrated diminished performances in contour detection compared with CTL. *No correlation was found between context processing and the different dimensions of the Schizotypal Personality Questionnaire*
Wang et al., 2013	SCZ (30), BD (13)	Binocular Depth Inversion Illusion	The SCZ group was less vulnerable to the illusion compared with both the CTL and BD groups (which were not different from each other). *A sub-group analysis suggested that this resistance was attributable to the 15 patients with the highest symptoms scores*
Yoon et al., 2009	SCZ and SCZ-T (17)	Surround suppression (Contrast discrimination)	The reduction of the surround-suppression found in SCZ (compared with CTL) was selective for stimulus orientation
**STUDIES THAT IDENTIFIED UNCHANGED, AUGMENTED OR CONTRASTED PATTERNS OF SENSITIVITY TO VIS IN SCHIZOPHRENIA OR PRO-PSYCHOTIC CONDITIONS**			
Chen et al., 2008	SCZ-T (24)	Surround suppression (motion discrimination)	The surround-induced bias was greater in patients compared with CTL. This was primarily because of a stronger inhibition, rather than a facilitation, effect
Chen et al., 2011	SCZ-T (33)	Spatial frame illusion	The illusory effect was greater for patients compared with CTL in visual, visuomotor and delayed visuomotor conditions
Kantrowitz et al., 2009	SCZ-T (38)	Surround suppression (size discrimination and Hermann grid illusion)	Patients showed different patterns of sensitivity depending on the illusion: increased for the Müller-Lyer illusion, unchanged for the Poggendorff illusion and Sander parallelogram, and decreased for the Ponzo illusion. These patterns depended on the contrasts of the stimuli
Norton et al., 2008	SCZ-T (28)	Three-flash illusion	The illusion peaked at a longer inter-stimulus interval in SCZ compared with CTL. At 100 ms, patients' vulnerability was decreased. In contrast, for higher intervals, patients perceived the illusion more frequently. *The three-flash illusion was primarily correlated with the positive and general subscale of the PANSS*
Tibber et al., 2013	SCZ and SCZ-T (24)	Surround suppression (discrimination of contrast, size, luminance and orientation)	Compared with CTL, patients were less biased by the context in their judgment regarding contrast and size but not luminance and orientation. *No correlation was found with the PANSS scores*
Yang et al., 2012	SCZ-T (30)	Surround suppression (discrimination of contrast, size, luminance, motion and orientation)	Patients exhibited more accurate (less biased) performances in contrast detection compared with CTL. However, the magnitude of the contextual modulation for luminance, size, orientation and motion was similar in both groups
Yang et al., 2013	BD (16), SCZ (30)	Surround suppression (discrimination of contrast, size, luminance, motion and orientation)	There was no difference in the surround effect influence between BD, SCZ and CTL groups for any task

*SCZ-T, treated schizophrenia; SCZ, untreated schizophrenia or unknown treatment status; FEP, first episode psychosis; IPS, initial prodromal state of psychosis; Δ9-THC, Δ9-Tetrahydrocannabinol; MDD, major depressive disorder; BD, bipolar disorder; CTL, controls; PANSS, positive and negative symptoms scale. The relevant clinical correlates are in italics*.

Second, several authors resorted to the structural approach to localize the specific disruption hypothetically responsible for the pattern of sensitivity to VIs observed in schizophrenia. However, these attempts led to apparent contradictions. This is notably the case when trying to assess, in a hierarchical perspective (from retina to high cortical areas), whether resistance to illusion is because of a high or a low-level disruption. For example, Norton et al. (2008) suggested an impairment of earlier visual processing by resorting to the *Three-flash illusion*. In this paradigm, a light pulse quickly presented twice appears as three flashes. The authors reported the illusion peaked to a longer inter-stimulus interval when presented to patients with schizophrenia compared with healthy controls. They argued that this temporal alteration may rely on a faulty sensory-integration within the very first stages of the visual stream. In contrast, by exposing participants to a battery of simple biasing-context illusions, Yang et al. (2012) and Tibber et al. (2013) found that luminance context processing was similar, whereas the strength of the illusions was inferior in patients for the other psychophysical parameters (e.g., contrast, orientation, size or motion). Thus, these authors hypothesized that the pre-cortical stages of the visual system, in which luminance is supposed to be processed, were spared, thereby contradicting Norton's conclusions.

VIs, although polymorphic, pinpoint the limits of purely anatomoclinical or linear causal models and underline, in contrast, the complexity and specificity of visual perception in schizophrenia. The data on VIs in schizophrenia patients encourage opting for a more functional and translational point of view.

### A window into the functional dimension of visual perception

VIs are an illustration of the perceptual system's ability to bind and group visual elements into coherent patterns, which leads to a meaningful representation. According to Butler et al. (2008) this phenomenon of *perceptual organization* can be viewed as relying on two main fundamental mechanisms: *gain control* and *integration*, both of which could be impaired in schizophrenia. Interestingly, studying VIs provides crucial elements for understanding these two elementary mechanisms.

*Gain control* can be defined as an adaptive process by which the sensory system optimizes information transmission to consider the visual context. For example, adjusting neural gains can concentrate the neuron's limited dynamical range around the mean contrast or luminance of the context, thereby ensuring that their responses do not saturate with luminance or contrast. *Gain control* of neural responses is achieved through a combination of intrinsic neuronal properties, lateral interactions and feedback modulations (Must et al., 2004).

**Figure 2:**
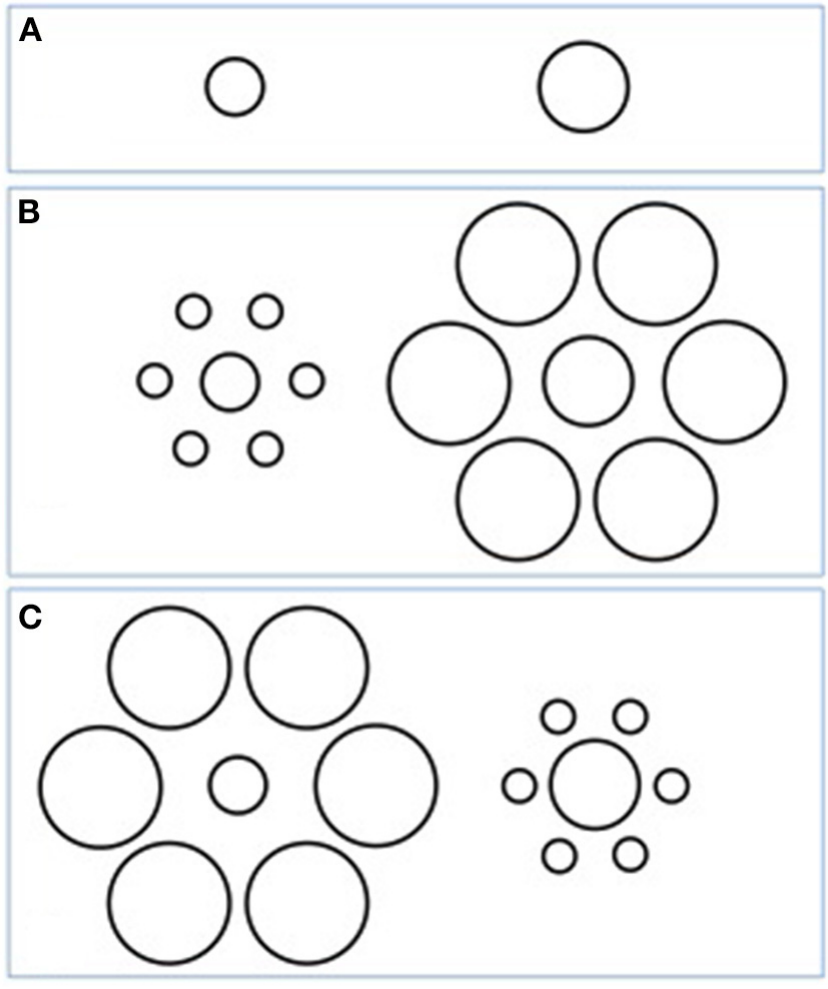
**An example of a context suppression effect: the Ebbinghaus illusion**. Depending on whether the peripheral circles are large or small, the central targets appear smaller or larger, respectively. When comparing two targets, the actual difference (here, left one smaller compared with right one) **(A)** appears reduced with the misleading context **(B)** or increased with the facilitating context **(C)**.

Although not always labeled as such, many VIs refer to a basic perceptual phenomenon called the *context* or *surround suppression effect*, which can be conceptualized as a form of *gain control*. This effect can be divided into facilitating or misleading effects when comparing two targets, depending on whether the context biases perception toward or away from the actual difference between the targets (see Figure 2). Some authors have argued, in line with the findings that stem from the study of VIs, that the impairment of *gain control* is a robust and significant property of visual processing in schizophrenia, and may even consider it a core feature of the disorder (Robol et al., 2013; Tibber et al., 2013). Nevertheless, carefully studying the VI literature encourages us to adjust and specify this hypothesis.

Despite the fact that the stimuli used in *surround suppression* VIs can easily be standardized, heterogeneous findings concerning schizophrenia have been reported in this sub-field (see Table 2). This noteworthy statement first raises the question of the interpretation of negative findings. Nevertheless, even if a lack of statistical power is a major cause of concern in neuroscience and may be imputed for several studies (Button et al., 2013), some clues suggest that heterogeneous findings in *surround suppression* may reflect, to a certain extent, the composite aspect of the underlying functional mechanism. (1) Some studies have probed *surround suppression* in different modalities through a battery of illusions in the same group of patients, thus limiting the methodological disparity. The authors failed to identify a steady impairment in the context effect across psychophysic characteristics. Furthermore, they could not reveal a constant inter-task correlation, which would have revealed a common uniform mechanism (Kantrowitz et al., 2009; Yang et al., 2012; Tibber et al., 2013). (2) Some evidence suggests that *surround suppression* and its impairment depends on several variables, such as the contrast (previously discussed) and the time of presentation (Calvert and Harris, 1988; Bressan and Kramer, 2013), which counters an absolute independent process. (3) The single phenomenon of contextual effect is not uniform and may be variously affected in its subcomponents as suggested by Chen et al. (2008). The authors proposed that context interaction impairment in schizophrenia may be better accounted for by alterations of surround inhibition rather than surround facilitation.

In complement to *gain control*, *integration* supports the visual system propensity to dynamically bind elements into complex perceptual constructs suitable for behavior or social skills (such as reading, face processing, and visual gnosis). From a neurophysiological point of view, *integration* relies on long-range projections of neuronal networks that connect superficial and deeper cortical layers. In VIs, the disruption in visual integration abilities becomes strikingly apparent when schizophrenia patients exhibit impaired coordinated visual skills necessary for the illusory effects to occur, such as stereopsis (Schechter et al., 2006). Recently, Schallmo et al. (2013) highlighted the abnormal integrative dimension of *perceptual organization* in schizophrenia, showing that patients' abilities for contour integration correlated with their *surround suppression* sensitivity, while these two illusory phenomena had previously only been shown to be impaired in isolation.

Unraveling such disruption of *perceptual organization* in its most basic mechanisms through VIs elucidates the connections with models that consider psychotic disorders a widespread deficit in cognitive coordination. However, testing this hypothesis and addressing how the VI literature may inform and be articulated requires a more quantitative framework, which is susceptible to coherently embrace all of the above-mentioned findings. Such computational theories should be able to integrate the results obtained in VIs and contribute new insight into the perceptual processes affected in schizophrenia. Among these theories, the Bayesian framework provides a natural framework for formalizing the interplay of integration, gain control, and feedforward and feedback processing in VIs in the general population and in schizophrenia.

## The computational point of view

### Visual illusions and the bayesian theory

The links between probabilistic theories and perception can be illustrated by examining a now famous VI example: the *Hollow-mask illusion* (readers can find a demonstration of the illusion on the following website: http://www.echalk.co.uk/amusements/OpticalIllusions/hollowMask/hollowface.html). In this VI, a face is presented as depth-inverted as the result of a pseudoscopic procedure (*Binocular depth inversion*). Despite this drastic counter-intuitive modification, healthy subjects still perceive the face as normally 3D-shaped, according to their knowledge of human anatomy (Gregory, 1973). This example nicely highlights how some VIs reveal the otherwise implicit interweaving between belief and perception by showing how prior expectations can overtake the actual objective properties of a given stimulus.

Starting from the Helmholtz's “unconscious inferences” theory (von Helmholtz, 1866), Bayesian models set uncertainty and belief as the core features of perception by considering sensory inputs as inherently ambiguous. In this probabilistic framework, perception is considered to result from optimal inferences concerning the world (see Box 1).

Box 1 **Bayesian theories of perception.**

The probabilistic approaches to perception consider sensory stimuli as inherently ambiguous. In order to build a coherent representation of the world, one has to combine uncertain sensory evidence with prior knowledge. Let us imagine that the task is to infer whether or not there is a tree (as summarized by a random binary variable theta). The Bayes theorem combines:

- **The prior:** the probability summarizing previous knowledge, before receiving the sensory information. For example, we may be in a forest, which implies a high prior probability for tree.
- **The likelihood**: the evidence provided by sensory organs. For example, what would be the probability of the observed retinal image given the presence of tree.

In order to compute:

- **The posterior**: the probability of the percept (is there a tree or not?) resulting from combining prior and likelihood.

**Table:** 

**Bayes theorem:**	p(θ|x) **Posterior**
$\text{p}\left(\theta |\text{x}\right)=\frac{\text{p}\left(\text{x}|\theta \right)\text{p}(\theta )}{\text{p}(\text{x})}$	=Probability of tree θ given the retinal input x
	p(x|θ) **Likelihood**
	= Probability of retinal input x given tree θ
	p(θ) **Prior probability for tree**
	= Probability of the parameter θ before any evidence

More generally, the Bayesian framework considers perception as a hierarchical inference process, with more abstract (higher) levels generating expectations and sending them down the cortical hierarchy (top-down process) toward sensory representation. Meanwhile, sensory evidence climbs up the hierarchy (bottom-up process) and activates these high levels representations. In this way, top-down expectations are constantly updated to account for new sensory evidence.

Several simplified framework have been proposed to describe hierarchical inference and relate it to the brain architecture.

- In **Predictive Coding** models, top-down predictions are subtracted from bottom-up inputs at each level of the hierarchy. The resulting prediction-error constitutes the input to the upper layer. This updates in turn the top-down predictions, which continues until the prediction errors are minimized. Each prediction error is weighted by an expectation regarding precision. This *gain of prediction errors* regulates the strength of the prior compare to sensory evidence, and is thought to be modulated by dopamine.
- The **Circular Inference** models see the inference process as multi-directional propagation of activity (beliefs) through excitatory feed-forward and feedback connections. To avoid a catastrophic reverberation of messages up and down the hierarchy (i.e., to avoid mistaking prior expectation for sensory evidence, or vice-versa), inhibitory loops must compensate excitatory loops in equal proportion, requiring a highly controlled excitatory-to-inhibitory balance. An imbalance will cause circular belief propagation and pathological inferences.

Importantly, despite conceptual differences, these frameworks are algorithmically similar and may essentially differ in the type of variables considered (e.g., binary vs. continuous, see Jardri and Deneve, 2013).

A re-examination, in the light of the Bayesian theory, of the stimuli that could have been traditionally considered illusory enables a conceptual refinement. Importantly, resorting to such a theoretical framework will help to re-delimit what does, and what does not fit with our definition of VIs.

In the Bayesian framework, the perceptual uncertainty that characterizes the “misleading” stimuli arises from weak or conflicting sensory evidence (Sundareswara and Schrater, 2008; Gershman et al., 2012), with 2 principal mechanisms.

1. *A significant dissociation between a strong expectation and the sensory evidence*. For example, the *Hollow mask* results from an imbalance between lower (the 3D-inverted stimulus) and higher (prior knowledge of anatomy) levels of abstraction. In this case, perception appears associated with a systematic bias toward prior knowledge. However, rather than a perceptual error, this bias is better understood as the consequence of inferences that optimally combine bottom-up and top-down information (see Boxes 1, **3**), which supports the best adaptation in a noisy environment. This corroborates, specifies and integrates in a theoretical framework the definition of illusions that we mentioned earlier (dissociation between the physical properties of the stimulus and the resulting percept). Importantly, several authors (Geisler and Kersten, 2002; Weiss et al., 2002) suggested that a whole range of illusory percepts, even those that are idiosyncratic in appearance (such as motion illusions), correspond to what an optimal Bayesian observer would perceive in similar conditions. This optimal systematic gap with sensory evidence could then also account for the illusory effect of figures for which the prior is not explicit or obvious. For example, the mechanism was proposed in different terms by Gregory when explaining the *Müller-Lyer illusion* (see Figure 1). According to this author, the internalized rules of perspective (i.e., prior knowledge) lead an individual to compute the line flanked with converging fins as if it were further away. Consequently, the line's shorter appearance conflicts with the actual identical size of the two lines.
2. *Inconclusive sensory evidence*. In ambiguous figures, nicely illustrated by the *Necker Cube* or *Rubin's vase*, perception switches between two mutually exclusive interpretations, a phenomenon called *bistability*. Although Bayesian formalism can be applied to such ambiguous figures, their inclusion in the field of visual illusions is more questionable and needs closer examination (see Box 2).

Box 2 **Do visual illusions comprise ambiguous figures?**

**Figure 3:**
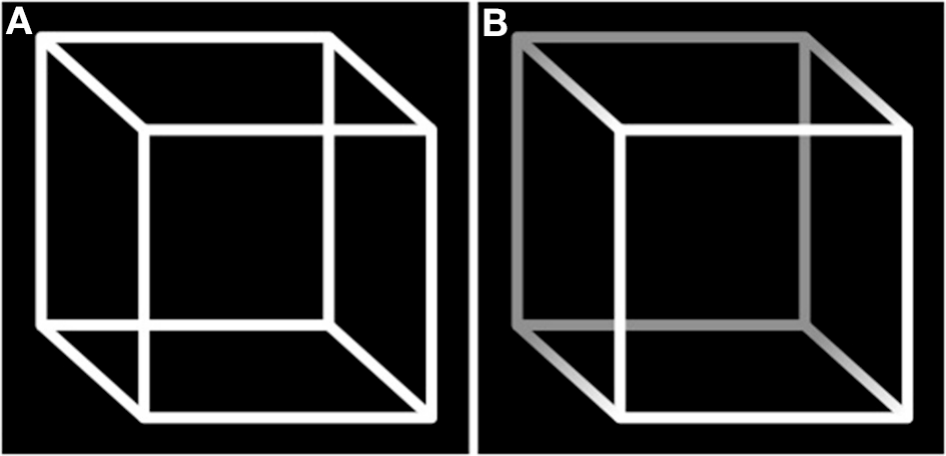
**Necker Cube**. **(A)** The ambiguity results in the subjective impression of two interpretations switching: the phenomenon of bistability. **(B)** The shadow introduces a cue that is supposed to bias perception toward one interpretation. The fact that bistability persists despite the presence of the cue ensures this cube responds to the definition of a visual illusion, i.e., a dissociation between perception and the physical characteristics of its support.

A figure can be considered ambiguous when it provides sensory information equally supporting different interpretations. This results in a phenomenon called *bistability*: in observers, two mutually exclusive percepts stochastically switch. An archetypal example is the Necker Cube, which is alternately perceived as if it was viewed from above and from below (see Figure 3A).

Assessing whether ambiguous figures (considered physical stimuli) and their subsequently generated percept are dissociated, i.e., whether ambiguous figures fit with the definition of illusions that we propose, requires dissociating two conceptual levels. We will use the Necker Cube as support for our demonstration.

- On the first level, the brain's propensity to derive a 3-dimensioned interpretation from a simple pattern of lines can be viewed as a basic discrepancy between sensation and perception. However, this discrepancy is inherent to the perceptual process, and thus irrelevant to specifying the ambiguous figure as an illusion.
- The second level refers to the question of whether bistability can be considered an illusory percept that arises from ambiguity. If we suppose a hypothetical perfectly ambiguous figure, the answer would be negative. Indeed, the information provided by the stimulus would be only sufficient to support the two equally probable resulting percepts. Thus, the image would equally coincide with the two interpretations. Nevertheless, if the bistable perception tended to persist despite the introduction of a cue (for example, by shadowing one corner of the Necker Cube, see Figure 3B), this would create dissociation between the stimulus properties and the induced percept: an illusion.

Note that we do not claim here that all perceptual illusions, without exceptions, fall in these categories. Rather, we propose a framework that can be applied to most of the perceptual illusions considered in this review.

### Visual illusions, bayes and psychotic symptoms

A growing field of theoretical and experimental approaches have related psychotic features, such as hallucinations and delusions, to a general disruption in the inferential process (Friston, 2005b; Fletcher and Frith, 2008; Schmack et al., 2013). By relying on the Bayesian formalism, authors typically impute the positive symptoms of schizophrenia to an imbalance in the relative weight or precision attributed to the prior and sensory evidence, which, in turn, causes false inferences. However, within this Bayesian framework, each model brings its own nuances in the way the system can be affected. The predictions sometimes clash or can be incompatible with each other. Importantly, any computational model should be sufficiently quantitative to enable the confirmation or rejection of the hypothesis based on experimental results and should be biologically plausible in terms of its mechanisms. Thus, it is important to consider in greater detail the type of Bayesian models and the possible sources of impairment that lead to schizophrenia symptoms for each of these approaches.

For example, according to the *Predictive Coding* model (see Boxes 1, 3) of hallucinations and delusions, the system is altered in its metacognitive components, i.e., in the estimates of the beliefs' precision rather than in the beliefs' precision itself (Adams et al., 2013). From this point of view, a disruption in these estimates results in the allocation of an insufficient or excessive gain in prediction errors. Notably, delusions, which can be defined as false and inflexible cognitive beliefs, are thought to originate from an excessive gain, which indicates attributing too much confidence to sensory evidence compared with prior beliefs. Artificially over-trusted, sensory signals become over-salient and unpredictable. They could be transmitted up the hierarchy, but no adjustments could fully resolve the aberrancy (Fletcher and Frith, 2008). Failing to adapt, the only solution for the system to explain away the erroneous prediction errors and resulting chaotic sensory signals is to generate aberrant beliefs at the top of the hierarchy. Because they constitute the only way to make sense of lower level sensory phenomena, these beliefs would progressively become inflexible and impervious to contradictory evidence (Schmack et al., 2013). Thereby, delusions could be avatars of tenacious prior beliefs secondarily generated by the system to restore coherence in the perceptual world (Adams et al., 2013).

Box 3 **Predictive coding and Circular inference.**

**Predictive coding**

Predictive coding applies the Bayes rule while assuming that the prior and the likelihood have a Gaussian distribution. For example, if the prior has mean $\hat{\theta }$ and variance σ^2^_θ_, and if the likelihood has mean x and variance σ^2^_x_, then the posterior is a Gaussian distribution with mean $\hat{\text{x}}$ provided by

$\hat{\text{x}}=\hat{\theta }+K\left(\text{x}-\hat{\theta }\right)$ with $K=\frac{\sigma_{\theta }^{2}}{\sigma_{\theta }^{2}+\sigma_{\text{x}}^{2}}$ corresponding to the Kalman gain.

**Figure 4:**
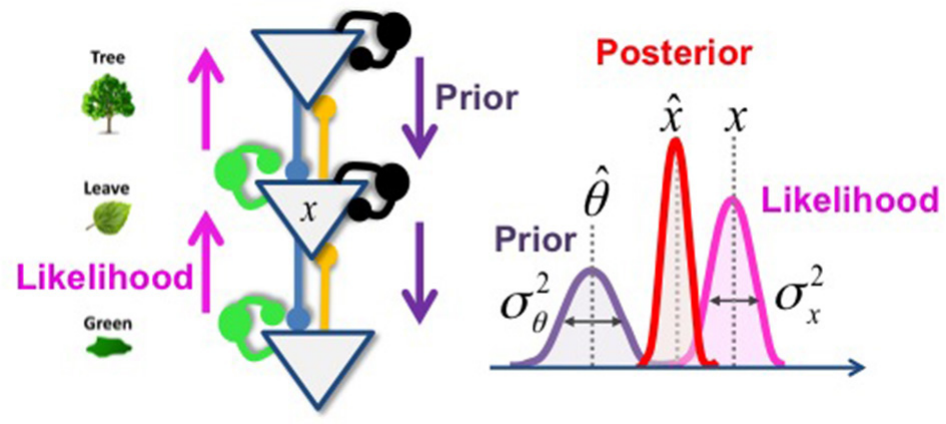
**Hierarchical inference with Gaussian variables**. In this toy example (left part), the inference that corresponds to the hidden cause (x) could be understood as the probability that the green color that I am observing (sensory evidence, represented by the magenta arrows) is due to the presence of a leaf, given my knowledge of the existence of a tree (prior expectation, represented by the violet arrows). Blue and yellow lines fit for the feedback and feedforward connections that enable the inferential process. Green and black circles fit for the controlling inhibitory system. The right part of the figure represents the probability distribution of each variable and the resulting posterior probability (in red).

Thus, the percept corresponds to the prior belief, corrected by a prediction error that corresponds to the difference between the sensation and its top-down prediction. The “salience” (Kalman gain) of the prediction error is a function of prior and sensory reliabilities. In a hierarchical network, this operation is repeated once for each layer, as schematized below (Figure 4):

**Circular inference**

**Figure 5:**
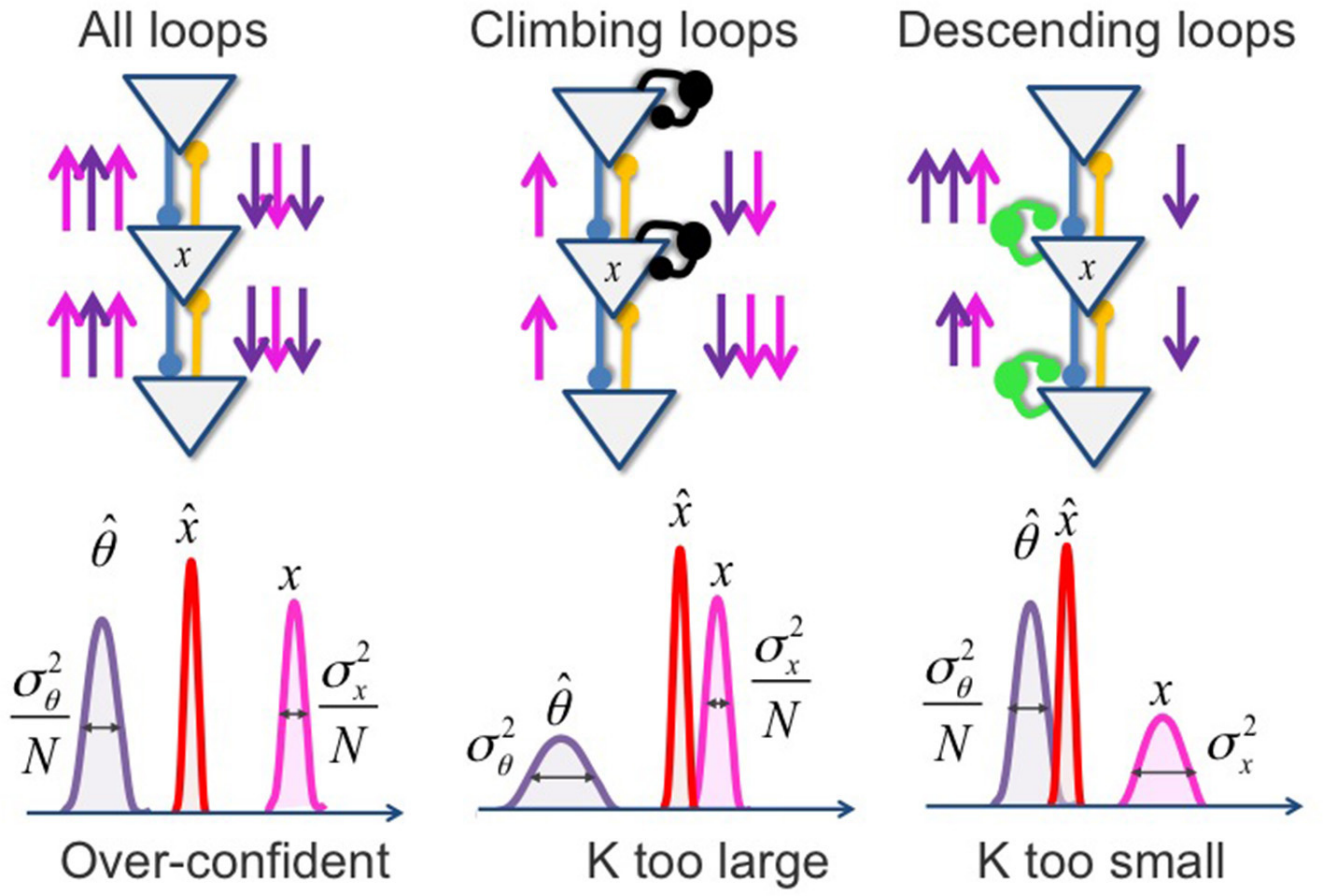
**Circular inference and relationship with predictive coding**. If both descending and climbing loops are impaired **(A)**, both sensory evidence and prior knowledge are reverberated and over counted (multiplication of the pink and violet arrows). In the *Predictive coding framework*, this results in an overconfident (narrowing of the posterior distribution) but not biased (unchanged K) inferred belief. In contrast, when the impairment only affects climbing loops **(B)**, sensory evidence, but not prior knowledge, is reverberated. The prediction error is emphasized (K is too large), and the inferred belief is biased toward sensory evidence. If, in contrast, the inhibitory disequilibrium disfavors the descending loops **(C)**, only the prior knowledge is over counted because of its reverberation. The prediction error is then minimized (K is too small), and the resulting posterior is biased toward expectations. Note that case **(B,C)**, the inferred belief is associated with an excessive degree of confidence.

To properly compute the probability of perceptual variables, the prior and likelihood must be multiplied only once. In the brain's hierarchy, top-down and bottom-up beliefs should be propagated only once in each direction (see figure above). This can be achieved if equally strong inhibitory loops exist to cancel excitatory feedforward/feedback loops (green and black units). If these inhibitory loops are impaired, beliefs are propagated multiple times, or, equivalently, the prior and likelihood are multiplied multiple times. The result is illustrated below (Figure 5):

In contrast, the hypotheses that have been proposed for the emergence of hallucinations in reference to the *Predictive Coding* model are not as univocal. As for delusions, some authors related misperceptions to an inferential disequilibrium that favors sensory evidence. According to this assumption, the emergence of maladaptive percepts is closely linked to failures in the system of self-monitoring, i.e., the ability to correctly identify oneself as the source of one's own actions and thoughts. When one plans an action, one predicts its sensory consequences upon an “efference copy” of this plan sent to the sensory cortical areas. When the action is achieved, the actual associated sensations are compared with the expectations. If they match (i.e., no prediction error), the action is labeled as “self-generated,” and the resulting sensations are attenuated. Conversely, a disrupted inferential system may fail to attenuate the sensory consequences of self-generated acts. Artificially overweighed, the resulting prediction error would then drive otherwise silent percepts to emerge into awareness. The only way to account for this unusual experience is then to misattribute it to an external agent. A key example is the delusional ideas regarding agency that arise from insufficient attenuation of the proprioceptive consequences of one's own movement (see Section What are the Links With Action Control and Motor Behavior?). Several authors have also related auditory hallucinations to an over-saliency of the inner speech (considered a covert motor action), which is misattributed to an alien source (McGuire et al., 1995; Allen et al., 2007; Moseley et al., 2013). However, in opposition to a theory of over salient sensory evidence, some authors have argued that excessively precise prior expectations, which correspond to a smaller-than-normal gain for prediction errors, better account for hallucinatory experiences. According to this hypothesis, perception distances itself from the sensory stimuli and becomes dependent on prior knowledge of the world (Friston, 2005b; Chambon et al., 2011; Schmack et al., 2013); thus, one would only perceive what one is expecting to perceive.

Note that the conflict between apparently contradictory hypotheses (gains of prediction errors larger than normal or smaller than normal, over-trusted prior or over-counted sensory evidence) has never been fully resolved, which renders the experimental testing of these theories extremely difficult. Finally, the neurophysiological processes that cause this imbalance remain unclear.

The *Circular Inference model* (see Box 1) attempts to overcome the contradictions by relating psychotic symptoms to a distributed excitatory-inhibitory imbalance (Jardri and Deneve, 2013). Inference in a hierarchical Bayesian system can be seen as a propagation of “bottom-up” messages (carrying sensory information) and “top down messages” (carrying top-down expectations). Posterior probabilities (and thus, percepts) are result of combining these two messages. Since long-range connections in the brain are overwhelmingly excitatory, these two types of messages would be reverberated endlessly through feed-forward/feedback excitatory loops if they were not controlled, and kept in check, by the presence of equivalently strong inhibitory loops. Indeed such balance is tightly maintained in cortical networks, and appears to be affected in schizophrenia (O'Donnell, 2011). In their model, Jardri and Denève showed how excitatory/inhibitory imbalance renders the system unable to avoid circular propagation of beliefs: bottom-up sensory evidence are reverberated back down as if they were prior information (upward loops), and thus combined with themselves until weak sensory inputs or meaningless coincidences are attributed to highly trusted high-level interpretations. Vice versa, prior expectations can generate their own “fake” sensory represents, which then come back up and reinforce the prior expectations (downward loops), in the absence of any true corroborative sensory evidence.

Psychotic manifestations can be understood as resulting from such circular inferences, which cause overconfidence, surinterpretations of weak sensory data and dissociations between high-level and low-level representations. This would be aggravated by an asymmetric impairment predominantly affecting either the upward or downward loops. Depending on which loops are mostly impaired, the model predicts that either sensory information or priors will dominate the final percept. This assumption is in line with the idea that hallucinations and delusions are two sides of a same coin (Fletcher and Frith, 2008). Even when facing weak or non-existent sensory evidence, the circular propagation generates strong perceptual beliefs: hallucinations occur where nothing relevant should have been inferred. In the same way, circular inference introduces spurious correlations between sensory (feedforward) and prior (feedback) messages that are non-existent in the real world. This leads to the learning and consolidation of “unshakable” (but false) causal relationships, resulting in delusional belief systems.

The *Circular Inference model* reconciles two eventualities that could have appeared to conflict in the *Predictive Coding framework:* hallucinations may stem from an overweighting of either prior or sensory evidence. Thus, asymmetrically impairing the inhibitory loops biases the inferential system in two opposite ways that both may generate the same aspecific perceptual phenomenon. This raises the interesting challenge of assessing which one of the two models is the most suitable to account for the emergence of hallucinations, which may depend on the disease that is related to these symptoms, and possibly on a subject-per-subject basis. Regarding schizophrenia, the question is still not clear. The reference to VIs, however, will help to clarify the issue. Indeed, a deficit in the control of upward loops (e.g., sensory evidence that reaches a high level representation and is then misinterpreted as prior knowledge when it is reverberated back down) would cause an over-confidence in sensory evidence and a lower susceptibility to perceptual illusions. Vice-versa, a deficit in the control of downward loops (e.g., prior expectations that activate lower levels and are misinterpreted as sensory information when they are reverberated back up the hierarchy) would predict an over-confidence in the prior knowledge and a higher susceptibility to perceptual illusions. Here we review three lines of relevant findings.

We previously discussed that schizophrenia might be primarily associated with a lack of sensitivity to VIs (see Section Discussing the Limits of the Structural Approach). Several authors have empirically interpreted this phenomenon as a sign of a reduced top-down influence in perception. Some representative examples support this assumption. (1) In *context suppression* illusions, the patients, who rely more on the absolute properties of the stimulus, tend to resist the perceptual bias induced by prior belief influence. (2) Top-down expectations are thought to be primarily responsible for the *Apparent motion illusion*, in which two stationary stimuli alternatively flickering induce an impression of movement. Resistance to the illusion in schizophrenia patients was notably attributed to their incapacity to correctly use top-down processes in this situation (Sanders et al., 2013). (3) The fact that patients with schizophrenia failed to correct for the inverted *Hollow Mask* to a more plausible (or predictable) interpretation can also be explained by an underweighting of prior knowledge during perceptual inferences (Schmeider et al., 1996; Schneider et al., 2002; Koethe et al., 2009).

Aside from these behavioral findings, recent brain-imaging and electrophysiology studies have complementarily supported the assumption of an overweighting of sensory evidence in schizophrenia. In two recent papers, Dima et al. explored the neural mechanisms involved in the resistance to the *Hollow mask illusion* via an event-related functional magnetic resonance imaging procedure (fMRI; Dima et al., 2009) and an event-related potential procedure (Dima et al., 2010). Using dynamic causal modeling (DCM), the authors notably showed a significant between-group difference in the effective connectivity patterns measured during the VI task. While a model that places connectivity modulation from the higher-level areas to the primary visual cortex (i.e., V1) better accounted for healthy control data, the DCM revealed a reverse pattern in schizophrenia patients (i.e., the predominance of the feedforward modulation). Overall, these findings are compatible with the idea of different perceptual strategies in individuals with schizophrenia and controls when trying to minimize strong prediction errors. When facing a VI (i.e., a complex perceptual task), a tendency toward a top-down counter-balancing is observed in controls, whereas patients who suffer from schizophrenia exhibit a strengthening of the bottom-up processes that prevent them from perceiving the illusory effect.

Interestingly, using magnetic resonance spectroscopy, Yoon et al. revealed that patients who suffer from schizophrenia exhibited a reduced GABA concentration in the visual cortex compared with healthy controls (Yoon et al., 2010). Furthermore, the authors found that this reduction predicted better (less biased) performances in the *surround suppression* task by observing that the lower rates of GABA observed in patients correlated with their tendency to be more accurate in contrast discrimination despite a misleading context. More recently, Loon et al. also supported the contribution of GABA in visual perception by focusing on *bistability*. With a computational model of the assumed neural underlying interactions, the authors first predicted that a higher GABA concentration in the visual cortex would result in a slowdown of the perceptual switches. More importantly, the authors then experimentally confirmed these predictions by observing, after the systemic administration of a GABA_A_ agonist (lorazepam), a lengthening of the percept durations and a decrease in the switch rates (Van Loon et al., 2013). Because GABA transmission reflects inhibitory neuronal modulations, these findings underpin the validity of circular probabilistic inference to account for perceptual impairments in schizophrenia by experimentally linking its assumed biological causes (excitatory-to-inhibitory imbalance) and the behavioral consequences (perceptual performances).

Overall, the heuristic value of VIs now appears clearer. Studying how patients cope with these simple stimuli provides access to underlying perceptual processes that could also account for the emergence of hallucinations and delusions (White and Shergill, 2012). In the case of schizophrenia, the evidence from the VI data converges toward the hypothesis of an asymmetrical belief formation that favors sensory evidence at the expense of prior knowledge. Among recently proposed models, the circular inference model appears particularly suitable to coherently link VIs, hallucinations, and their plausible biological causes. Indeed, schizophrenia resistance to VIs and susceptibility to hallucinations can be considered to result from the same circular inferential process in an ambiguous environment. This assumption has strong support in studies that identified negative correlations between illusion susceptibility and the presence of positive symptoms in healthy (Bressan and Kramer, 2013) and clinical populations (Keane et al., 2013; Sanders et al., 2013).

Importantly, while VIs provide a privileged access to the visual hallucinatory modality, readers should note that adult patients who suffer from schizophrenia are more concerned by auditory compared with visual hallucinations (Mueser et al., 1990; Blom, 2010). However, a recent review paper examined the clinical, phenomenological, psychological and physiological properties of visual hallucinations in schizophrenia compared with the same symptoms observed in Parkinson disease, Body-Lewy dementia or the Charles-Bonnet syndrome. The authors reported visual hallucinations with a substantial point prevalence of 27% (Waters et al., 2014). Mostly disregarded in the literature that examined hallucinations until recently, the visual modality is the subject of a renewed interest. Furthermore, we hypothesize that the demonstration we utilized for visual illusions and hallucinations could easily be transferrable to the auditory perceptual process. Moreover, this would be an interesting assumption to explore in future research.

Positive symptoms are not specific to schizophrenia. The possible occurrence of hallucinations and delusions in various psychiatric and neurological conditions or even in non-clinical populations suggests the relevance of a dimensional approach. Nevertheless, the heuristic value of VIs may open a path toward a new categorization based on the computational model that offers the best fit with particular perceptual disruptions. We effectively illustrated how an overweighting of sensory evidence may explain both hallucinations and the reduced susceptibility to VIs in schizophrenia. Interestingly, a trend to resist VIs has also been identified in autism (Happé, 1996), which suggests that an imbalance in processing toward sensory evidence could also offer a coherent and appropriate comprehensive framework for this developmental disorder (Pellicano and Burr, 2012). In contrast, some neurological disorders associated with hallucinations may exhibit an increased tendency to experience VIs. This tendency is noteworthy in the case of Parkinson's disease, in which one quarter of patients suffer from visual hallucinations and visual misperceptions (Diederich et al., 2009). Even if experimental validation is required, references to the *Circular Inference framework* (Jardri and Deneve, 2013) indicate this pattern potentially results from a reverse asymmetric impairment in the excitatory-inhibitory balance (i.e., an overweighting of prior information relative to the sensory evidence, which is caused by an insufficient inhibitory control of downward loops). This impairment could explain the concomitant increase in hallucinations and illusions in Parkinson's disease (as could the *Predictive Coding model*).

## Theoretical and clinical implications for schizophrenia research

### What are the links with psychopathology?

The resistance to VIs in schizophrenia patients leads to several etiopathological implications and may drive several new experiments. For example, it appears possible to examine the correlations between this lack of susceptibility and different clinical features. Despite an abundant literature dealing with this matter, frequent methodological issues (e.g., inter-group comparability) have made the findings difficult to interpret. Three main approaches may be individualized:

- Examining the potential links between VI sensitivity and symptom severity (primarily using the PANSS scale) first provided discrepancies in the findings because these scores were computed as covariates. While some authors found no or only weak relationships between VIs and psychopathology (Koethe et al., 2006, 2009; Tibber et al., 2013; Yang et al., 2013), other authors identified an inverse correlation with either positive (Norton et al., 2008; Keane et al., 2013; Silverstein et al., 2013) or negative (Tadin et al., 2006; Silverstein et al., 2013) symptom dimensions. To draw valid conclusions, designs specifically focusing on the clinical correlates of VI insensitivity in clinical populations, with more precise and specific symptom assessments, appear necessary.
- A second question frequently raised by the resistance to VIs in schizophrenia is whether this property could be considered a trait or a state marker of the disorder. Using the *Binocular depth inversion* paradigm, Koethe et al. (2009) identified a significant reduction in VI sensitivity not only in medicated schizophrenia patients but also in antipsychotic-naïve and prodromal state individuals. Thus, these authors considered their findings as indicative of a trait or early state characteristic within the schizophrenia spectrum. However, this assumption was not confirmed by longitudinal studies. In two experiments that prospectively assessed how clinical evolution impacts VI perception, a normalization of sensitivity to illusions was observed in the schizophrenia groups that received inpatient treatment, which became comparable to controls (Schneider et al., 2002; Silverstein et al., 2013). Even if further investigation is still required, at this point, we have identified stronger evidence for a state-dependent pattern of VI susceptibility in psychosis.
- A compelling question derived from previous findings would be whether resistance to VIs is exclusively related to schizophrenia. If so, this would represent an argument for a new nosographic individualization of this condition as a result of a perceptual property, which is easy to assess. In this sense, studies that directly compared patients who suffered from schizophrenia with patients who suffered from bipolar or major depressive disorders found that the latter two groups were normally sensitive to *Binocular depth inversion* (Schneider et al., 2002; Koethe et al., 2009; Wang et al., 2013) and *Contrast modulation* (Dakin et al., 2005; Yang et al., 2013), which was significantly different from the patients with schizophrenia. However, as previously discussed, in autism, subjects also exhibited a tendency to resist VIs (see Section Visual Illusions, Bayes and Psychotic Symptoms), which may contradict the specificity of this pattern. Interestingly, other studies have suggested that the induction of “pro-psychotic” states in non-clinical populations, via, for example, acute cannabinoids (Emrich et al., 1997; Leweke et al., 1999; Koethe et al., 2006), chronic intoxication (Semple et al., 2003) or sleep deprivation (Sternemann et al., 1997), enable to experimentally reduce sensitivity to VIs. In another experiment that utilized the *Ebbinghaus illusion*, Bressan and Kramer recently reported that the VI magnitude decreased with schizotypal traits in a naïve student population (Bressan and Kramer, 2013). Thus, these converging clues prompt one to refute the idea that resistance to VIs is a specific perceptual property of schizophrenia and, hence, an endophenotypic marker for psychosis in general. The progressive proneness to VIs may be better viewed as related to the vulnerability to specific clinical dimensions of the disorder (e.g., positive symptoms) via a progressive disruption in the inferential processes that underline perceptions and cognitions. According to this hypothesis, the more weight that is attributed to sensory evidence (because of a spontaneous or induced variable disequilibrium in the excitatory-to-inhibitory balance), the less prone the subject is to VIs, and the more vulnerable he will be to delusional beliefs and hallucinations. Interestingly, the opposite profile of asymmetric circular belief propagation (selective impairment of upward loops) predicts opposite (increased) vulnerability to VIs but similar symptoms. Thus, the possible reverse pattern of dissociation between prior and sensory evidence leads one to consider the corollary proposal: the more weight that is attributed to prior knowledge, the more vulnerable the subject is to both VIs and hallucinations. This proposal may account for the gradual increase in susceptibility to VIs that accompanies the progressive emergence of misperceptions in some psychiatric or neurological conditions, such as Parkinson's disease (see also Section Visual Illusions, Bayes and Psychotic Symptoms).

### What are the relations with subjective experiences?

Considering the *Circular Inference* model, aberrant experiences in schizophrenia may be understood as an immersion in a world dominated by sensory evidence, while top-down influences lose their organizing and structuring potential. When the ambiguity is particularly strong, VIs represent an extreme perceptual task. As such, their study provides an emphasized insight into the phenomenology of perception. Although the hypothesis requires further investigation, we can assume that during the vast majority of daily life situations, patients resolve the moderate ambiguity of the environmental stimuli through strong and unambiguous percepts but with weakened links and coherence between perceptual elements (links typically implicitly driven by prior knowledge). The perceptual world would then appear as fragmented and less meaningful (Adams et al., 2013). Hallucinations would only emerge when facing highly uncertain (and normally irrelevant) sensory information. Importantly, because the necessity to adapt to highly ambiguous situations is not permanent, this may also account for the intermittent nature of hallucinations (Jardri et al., 2013).

This model may account for the cognitive deficits observed in schizophrenia, such as the patients' difficulties in correctly allocating attention and filtering out irrelevant information. This phenomenon can also be explained by the lack of prior influence on saliency. The weakening of the downward beliefs blurs the distinction between relevant and noisy items, which makes them almost equally surprising.

### What are the links with action control and motor behavior?

The question of whether the highlights provided by VIs help us understand behavioral features in schizophrenia requires one to consider both the complex links between perception and action and the possible common causes for their disturbances. In this regard, paradigms that test an illusory effect via visuomotor performances are thought to engage a complex cross-modal coordination and, thus, are of particular interest in schizophrenia (Pessoa et al., 2008; Chen et al., 2011).

Moreover, several lines of evidence suggest that the tendency to resist illusions observed in schizophrenia is not limited to the visual modality. Shergill et al., for example, studied the *Force matching illusion*, which consists of the systematic underestimation of a self-generated force deployed to match an externally applied target force (Shergill et al., 2005). In normal conditions, a system of self-monitoring enables the prediction of the sensory consequences of one's motor acts. In Bayesian terms, the predictions, which arise from prior knowledge, regarding the sensory outcome of one's own action permit a reduction in the weight attributed to the matching sensory evidence, and hence, attenuate the perception related to this sensation. The authors indicated that patients who suffered from schizophrenia were more accurate compared with controls when matching the externally applied force, which revealed a failure in the normal sensory attenuation mechanism. This finding outstandingly fit with the hypothesis of a false inferential process overtook by sensory evidence. Brown et al. (2013) went further in a recent paper by demonstrating how perception and action were derived from the same Bayes optimal system. Referring to the notion of *active inference*, the authors considered movement as a way to actively minimize proprioceptive prediction errors. However, this process is conceivable only if it is combined with a reduction of precision of sensory evidence to avoid conflict between action and perception. Using a probabilistic generative model, the authors predicted that a failure in attenuating sensory proprioceptive evidence led to the emergence of delusional ideas regarding agency (the ability to correctly identify oneself as the cause of one's own actions), as well as a decreased susceptibility to the *Force-matching illusion*. These findings are in accordance with previous experimental reports of resistance to VIs in schizophrenia but extend this observation to multisensory perception.

Overall, the Bayesian framework predicts that false inferences, which are biased by overweighted or insufficiently attenuated sensory evidence, may coherently (1) account for both the visual and proprioceptive perceptual changes in schizophrenia, (2) closely link these changes with action, and by extension, behavioral disruptions, (3) explain the emergence of hallucinations and delusional beliefs, and (4) provide an heuristic value to the vulnerability to illusions, which can be considered an indirect but valuable access to the global neural processing in this disorder.

## Conclusion

Under the Bayesian scope, VIs acquire tremendous heuristic value by providing new insights into the perceptual processes that underlie misperceptions. The literature that pertains to VIs has paved the way for new hypotheses regarding psychiatric and neurological conditions (for example, overcounting of sensory evidence in schizophrenia vs. prominence of prior knowledge in Parkinson's disease). Moreover, through the probabilistic framework, VIs are an indirect but promising approach to understand several schizophrenia features as coherently emerging from the same inferential process. The research avenue may benefit from a more rigorous methodological approach, particularly by resorting to more precise classifications and conceptual definitions.

### Conflict of interest statement

The authors declare that the research was conducted in the absence of any commercial or financial relationships that could be construed as a potential conflict of interest.
